# COVID-19-related acute macular neuroretinopathy in China: a retrospective study of 90 cases (152 eyes)

**DOI:** 10.1186/s40942-026-00825-2

**Published:** 2026-02-28

**Authors:** Hua Fu, Boya Chen, Yanming Xu, Houyue Liu, Zeyu Cai, Qiannan Chai, Nalei Zhou, Xuejing Li, Jialiang Duan

**Affiliations:** Department of Ophthalmology, The Second Hospital of Hebei Medical University, No. 215, West Heping Road, Shijiazhuang City, Hebei Province 050000 People’s Republic of China

**Keywords:** Coronavirus disease 2019, Acute macular neuroretinopathy, Microvascular, Thrombosis, Infection

## Abstract

**Background:**

Acute macular neuroretinopathy (AMN), a rare retinal disorder, is characterized by mild visual impairment that is associated with macular lesions. AMN cases increased during the coronavirus disease 2019 (COVID-19) pandemic. Chinese ophthalmology clinics have also observed an increase in cases since the end of 2022. We analyzed the clinical and epidemiological characteristics of COVID-19-related AMN cases in China.

**Methods:**

This retrospective study included 90 patients (152 eyes). Information regarding COVID-19-related AMN cases was collected from online consultations and local clinics. Cases were diagnosed using multimodal fundus imaging. Statistical analyses were performed to evaluate clinical features and prognostic characteristics.

**Results:**

Most patients were female (*n* = 70; 78%) and young (mean age, 28.6 ± 6.0 years). Bilateral AMN lesions were observed in 69% (*n* = 62) of patients. Most patients (*n* = 79; 88%) experienced a peak in ocular symptoms on the second to third day after COVID-19 symptoms, following a high fever. The most common ocular symptom was scotomas (*n* = 63 eyes; 41%). Most eyes (*n* = 112; 74%) had a baseline best-corrected visual acuity of ≥ 20/40. Among 56 patients (96 eyes) with follow-up durations of 3–15 months, changes in patient-reported visual symptoms were observed, with improvement in 41% of eyes (*n* = 39), no change in 46% (*n* = 44), and worsening in 13% (*n* = 13).

**Conclusions:**

Recognizing and addressing COVID-19-related retinal complications, particularly AMN, are important. Follow-up is crucial for monitoring the progression and evolution of visual symptoms and retinal changes over time.

**Supplementary Information:**

The online version contains supplementary material available at 10.1186/s40942-026-00825-2.

## Background

Acute macular neuroretinopathy (AMN), a rare retinal disorder first described by Bos and Deutman in 1975 [1], is characterized by the acute onset of mild visual impairment and is often associated with lesions within the macula. These lesions typically exhibit irregular hyperreflectivity in the outer plexiform layer and outer nuclear layer, as visualized using optical coherence tomography (OCT).

From 1975 to 2019, approximately 150 AMN cases were documented. However, the number of diagnosed AMN cases sharply increased after the global coronavirus disease 2019 (COVID-19) pandemic, and the correlation between viral infections and AMN attracted much attention [2, 3]. Ophthalmology clinics in China experienced a notable surge in patients presenting with AMN following the rapid escalation in confirmed COVID-19 cases since the end of 2022 [4].

In this study, we compiled data obtained through online consultations of patients diagnosed with AMN across 47 cities in China and from our local eye clinic, which comprised a total of 152 eyes from 90 patients. Based on these data, we analyzed the clinical and epidemiological characteristics of AMN after COVID-19. This descriptive study focused on epidemiological findings, including case characteristics categorized by patient age, sex distribution, pathogenic factors, and ocular symptoms prognoses.

## Methods

### Data collection

This study included 152 eyes of 90 patients, whose data were obtained from online consultations and our eye clinic. The patients, recruited from 47 cities across China, were initially diagnosed with COVID-19 and subsequently diagnosed with AMN between December 2022 and February 2023. Data collection primarily relied on an internet survey comprising an online consultation and structured questionnaires (patient Nos. 1–85), supplemented by five cases (patient Nos. 86–90) diagnosed at our eye clinic. All patients tested positive for COVID-19 after developing influenza symptoms. Patients who were followed up for more than 3 months were included in the follow-up group in this study. Follow-up information was collected from imaging examinations performed at our ophthalmology clinic, and from online submissions of medical records from local medical institutions.

### Ethical statement

This study was approved by the Second Hospital of Hebei Medical University (no. 2024-R544). The study adhered to the tenets of the Declaration of Helsinki. Written or electronic informed consent for publication of identifying information (including images and other personal or clinical details) was obtained from all participants (or their legal guardians), and efforts have been made to anonymize such details wherever possible.

### Diagnostic criteria

**Fig. 1:**
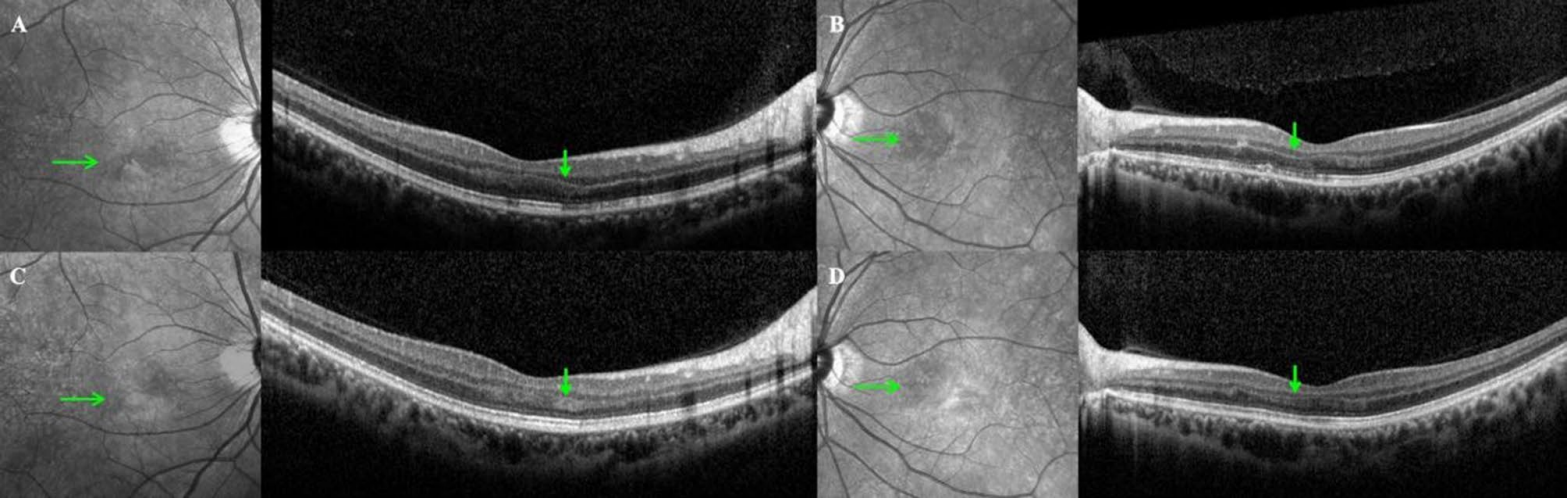
Representative OCT findings of acute macular neuroretinopathy. Patient No. 90 (**A**–**D**): a 25-year-old woman with blurred vision in both eyes and a positive COVID-19 test 1 day earlier. At the first visit, the initial BCVA was 20/30 in the right eye (OD) and 20/100 in the left eye (OS). From the first OCT examination (**A**, **B**), parafoveal hyporeflective lesions on NIR and outer retinal hyperreflectivity with EZ disruption on SD-OCT are observed in both eyes (green arrows). Follow-up OCT (**C**, **D**) at 15 months shows thinning of the outer nuclear layer and partial EZ loss. The BCVA was 20/30 in both eyes at the final follow-up. OCT, optical coherence tomography; COVID-19, coronavirus disease; NIR, near-infrared imaging; EZ, ellipsoid zone; SD-OCT, spectral-domain optical coherence tomography; BCVA, best-corrected visual acuity

COVID-19-related AMN was diagnosed based on the following criteria [5]: (1) positive antibody serological test or COVID-19 antigen test (on nasopharyngeal swab or oropharyngeal swab); (2) a history of acute-onset paracentral or central scotoma, with or without a decrease in visual acuity, emerging immediately after a positive COVID-19 test; (3) Amsler grid tests showing visual disturbances; (4) parafoveal hyporeflective lesions observed on near-infrared reflectance imaging; and (5) hyperreflectivity of the outer plexiform layer/outer nuclear layer junction accompanied by attenuation of the underlying ellipsoid zone (EZ) on spectral-domain OCT (SD-OCT) (Fig. 1) or swept-source OCT angiography.

### Exclusion criteria

Throughout the screening and differential diagnosis processes, certain data were excluded based on the following criteria: (1) incomplete interviews: uploaded ophthalmic imaging data but no response to the online interview; (2) low-quality ophthalmic imaging: these were excluded to ensure data veracity and precision; (3) non-consent for data contribution: refusal to provide personal data for research purposes, in adherence to ethical standards and the safeguard of individual privacy; or (4) considerations for a differential diagnosis: presenting with conditions such as punctate inner choroidopathy, multiple evanescent white dot syndrome, acute zonal occult outer retinopathy, or typical optic neuritis and other retinal diseases. The imaging data of all patients were analyzed by four readers (QC, NZ, XL, and JD), with the fourth reader (JD) making the final decision in cases of disagreement.

## Results

### Epidemiological characteristics

Of the 90 patients (152 eyes) diagnosed with COVID-19-related AMN included in this study, 70 (78%) were females. The mean age of the patients was 28.6 ± 6.0 (range, 14–48) years. Bilateral involvement was exhibited in 62 patients (69%), and unilateral involvement was predominantly observed in the left eye (among the 28 unilateral cases, 20 were in the left eye). In addition to COVID-19, other potential concurrent risk factors for AMN documented in patients’ medical histories included concurrent or recent use of contraceptive medications (both long and short-acting), recent Cesarean section, and ocular hypertension. Baseline systemic diseases included hypertension, kidney disease (chronic nephritis or kidney failure), anemia, migraine, myocardial ischemia, and bradycardia (Table 1). Further details regarding patient medical histories are in the Supplementary File.

**Table 1:** Patient epidemiological characteristics

Feature	Patients (*n* = 90)			Patients (%)
**Sex**				
Males	20			22
Females	70			78
**Age at presentation**,** years**				
14–17	2			2
18–39	86			96
40–48	2			2
Mean age ± standard deviation, years	28.6 ± 6.0			
**Bilateral or unilateral involvement**				
Bilateral (OU)	62			69
Unilateral	28	OD	8	31
		OS	20	
**Concurrent risk factors of acute macular neuroretinopathy**				
Contraceptive medication	5			6
Ocular hypertension	3			3
Recent cesarean operation	1			1
**Baseline systemic disease**				
High blood pressure	8			9
Kidney diseases (chronic nephritis or kidney failure)	4			3
Anemia	3			3
Migraine	3			3
Myocardial ischemia, Bradycardia	1			1

OD, oculus dexter; OS, oculus sinister; OU, oculus uterque

Almost all patients (*n* = 89; 99%) were affected by COVID-19 for the first time, with only one patient (No. 85) diagnosed with AMN during the second infection. Ocular symptoms commenced on the second to third day following influenza symptoms (including fever, sore throat with cough, and headache) in most patients (*n* = 79; 88%). Furthermore, ocular symptoms consistently manifested after an increased temperature; 68 patients (76%) had a high fever (≥ 38.6 °C), 13 (14%) had a mild fever (37.5–38.5 °C), and nine (10%) reported an increase in body temperature but without the exact degree. In addition, 41 patients (46%) reported profuse diaphoresis secondary to high fever. Overall, 67 patients (74%) confirmed they had been vaccinated against COVID-19; six had post-vaccination side effects (skin rashes, fever, fatigue, headache, episodic hypertension, conjunctivitis, and painful edematous plaques in the injection site) (Table 2 and Supplementary File).

**Table 2:** Possible influencing factors for COVID-19-related acute macular neuroretinopathy

Feature	Patients (*n* = 90)	Patients (%)
**First infection with COVID-19**		
True	89	99
False	1 (patient No. 85)	1
**Influenza-like symptoms to visual symptoms interval**		
Day 1	9	10
Day 2	50	56
Day 3	29	32
Day 4	2	2
**Fever degree after COVID-19 infection**		
Normothermia (35.6–37.4 °C)	0	0
Mild fever (37.5–38.5 °C)	13	14
High fever (≥ 38.6 °C)	68	76
Unchecked	9	10
**Profuse diaphoresis secondary to high fever**		
True	41	46
False	49	54
**COVID-19 vaccinated**		
True	67	74
False	23	26

COVID-19, coronavirus disease 2019

### Ocular symptoms in COVID-19-related AMN

The cohort exhibited diverse ocular symptoms, which included scotomas in 63 eyes (41%), mosaic blurred vision in 54 eyes (36%), scintillating scotomas in 31 eyes (20%), and black dots in four eyes (3%). Most eyes (*n* = 112; 74%) had a baseline best-corrected visual acuity (BCVA) of ≥ 20/40 (Table 3).

**Table 3:** Ocular symptoms with COVID-19-related-AMN

Feature	Eyes (*n* = 152)	Eyes (%)
**Baseline BCVA of COVID-19-related AMN**		
20/20–20/40	112	74
20/50–20/100	16	10
20/200–20/400	20	13
Unknown	4	3
**Symptoms**		
Scotomas	63	41
Mosaic blurred vision	54	36
Scintillating scotomas	31	20
Black dots	4	3

AMN, acute macular neuroretinopathy; BCVA, best-corrected visual acuity; COVID-19, coronavirus disease 2019

### Follow-up data

Patients who were followed up for more than 3 months were included in the follow-up group in this study. Follow-up data were available for 56 patients (96 eyes), including current BCVA, additional symptoms reported during follow-up, changes in visual symptoms, and OCT findings.

The mean follow-up duration was 9 (range, 3–15) months. Among the 96 eyes included in the follow-up cohort, final BCVA was at ≥ 20/40 in 83 eyes (86%), whereas 13 eyes (14%) had a final BCVA between 20/50 and 20/200. All patients underwent OCT examinations at each follow-up visit. A chi-square analysis demonstrated a significant association between initial OCT foveal involvement and final BCVA outcome (χ² = 4.53, *P* = 0.033). Initial OCT demonstrated foveal center involvement in 100% of these 13 eyes with BCVA between 20/50 and 20/200, compared with 76% foveal involvement among eyes with final BCVA between 20/20 and 20/40 (Table 4). Within the first 2 months of the follow-up period, 80 eyes (83%) experienced new ocular symptoms, such as water ripples (46 eyes) and frequent ocular fatigue (24 eyes). Floaters (24 eyes) and visual snow (six eyes) gradually manifested after 2 months (Table 5). During follow-up, patient self-reports indicated that obstruction gradually became transparent or faded in 39 eyes (41%, classified as “improved”), remained unchanged in 44 eyes (46%, classified as “unchanged”), and worsened in 13 eyes (13%, classified as “worse”).

**Table 4:** Chi-square analysis of initial foveal involvement and final BCVA outcome

	Category	Fovea involved, *n*	Fovea not involved, *n*	Total, *n*	χ²	*P*
Final BCVA	20/20–20/40	63	20	83	4.53	0.033*
	20/50–20/400	13	0	13		
Total		76	20	96		

BCVA, best-corrected visual acuity

**Table 5:** Follow-up data

Features	Eyes (*n* = 96)	Eyes (%)
**Best-corrected visual acuity (follow-up)**		
20/20–20/40	83	86
20/50–20/100	12	13
20/200–20/400	1	1
**Patient-reported changes in visual symptoms**		
Improved	39	41
Unchanged	44	46
Worse	13	13
**Additional symptoms during follow-up**		
Water ripples	46	
Frequent ocular fatigue	24	
Floaters	24	
Visual snow	6	
No secondary ocular symptoms	16	
**Initial OCT characteristics-AMN lesions involved the foveal center**		
True	76	79
False	20	21

Additional symptoms during follow-up were recorded per eye and were non–mutually exclusive; multiple selections were permitted, and total counts exceed the number of eyes

## Discussion

Since it was first reported in 1975 [1], AMN has been a rare disease outlined in a few case reports; however, its pathogenic factors and pathological mechanism have always been of interest and controversial. Previous studies have reported cases of AMN after a viral infection or influenza symptoms. Turbeville et al. [6] summarized the potential risk factors of AMN based on 41 cases reported until April 2002. Among these cases, 18 (44%) had a history of an influenza-like syndrome before the ocular events. Bhavsar et al. [5] reviewed 156 eyes from 101 cases reported until December 2014 and 48 (47.5%) patients developed ocular symptoms of AMN after infection or febrile illness, which is the approximate proportion reported by Turbeville et al. [6] Furthermore, Li et al. [7] reported five patients (nine eyes) with AMN associated with dengue fever from a mosquito-transmitted viral epidemic in South China in 2014.

Since the onset of the COVID-19 pandemic, the number of confirmed cases of AMN has rapidly increased [8, 9]. For instance, in a French hospital, Azar et al. [10] found a significant increase in AMN cases diagnosed during the 2020 COVID-19 pandemic compared with those of other retinal conditions, such as paracentral acute middle maculopathy and multiple evanescent white dot syndrome. Chinese ophthalmology clinics also observed an increase in the number of patients with AMN concurrent with a rapid rise in confirmed COVID-19 cases since the end of 2022 [11, 12].

To the best of our knowledge, the information collected in this study represents the largest group of COVID-19-related AMN cases to date. Most COVID-19-related AMN cases had various similarities in patient features and ocular symptoms with previously reported AMN cases without COVID-19: a higher proportion of females (*n* = 70; 78%); a younger demographic with a mean age of 28.6 ± 6.0 years; and diverse ocular symptomatology, including central or paracentral scotomas, mosaic blurred vision, scintillating scotomas, and black dots. We analyzed the case data and obtained the following significant characteristics: (1) bilateral lesions in 62 patients (69%), which was higher than previously reported rates [5]; (2) COVID-19 recorded for the first-time in 89 patients (99%); (3) ocular symptoms occurring after high fever (≥ 38.6 ℃) in 68 patients (76%), with the peak of the ocular symptoms starting between the second and third day following influenza symptoms in 79 (88%) patients, with an exception observed in a 24-year-old woman (patient No. 10), whose symptoms manifested 4 days before she had a fever, and her ocular symptoms worsened after a high temperature of 39 °C, when COVID-19 was confirmed; (4) the incidence of AMN causing serious visual impairment was relatively low with most eyes (*n* = 112; 74%) having a BCVA of ≥ 20/40, 10% of the eyes (*n* = 16) having a BCVA between 20/50 and 20/100, and 13% (*n* = 20) having a low BCVA (≤ 20/200); and (5) high prevalence of additional ocular symptoms during follow-up. A substantial number of eyes (*n* = 80; 83%) had secondary ocular symptoms, including water ripples, frequent ocular fatigue, floaters, and visual snow. (6) A chi-square analysis revealed a significant association between foveal involvement in initial OCT and final visual outcomes. This association may be partially explained by the fact that foveal involvement selectively disrupts the highest-density cone photoreceptors and the central visual pathways responsible for maximal visual acuity, thereby exerting a disproportionate impact on visual function. (7) Changes in patient-reported visual symptoms during follow-up were markedly heterogeneous. Improvement was reported in 39 eyes (41%), no change in 44 eyes (46%), and worsening in 13 eyes (13%). Importantly, a favorable follow-up BCVA did not necessarily indicate improvement in visual field obstruction. Among the 13 eyes in the “worse” group, 7 eyes still achieved a BCVA of 20/20.

**Fig. 2:**
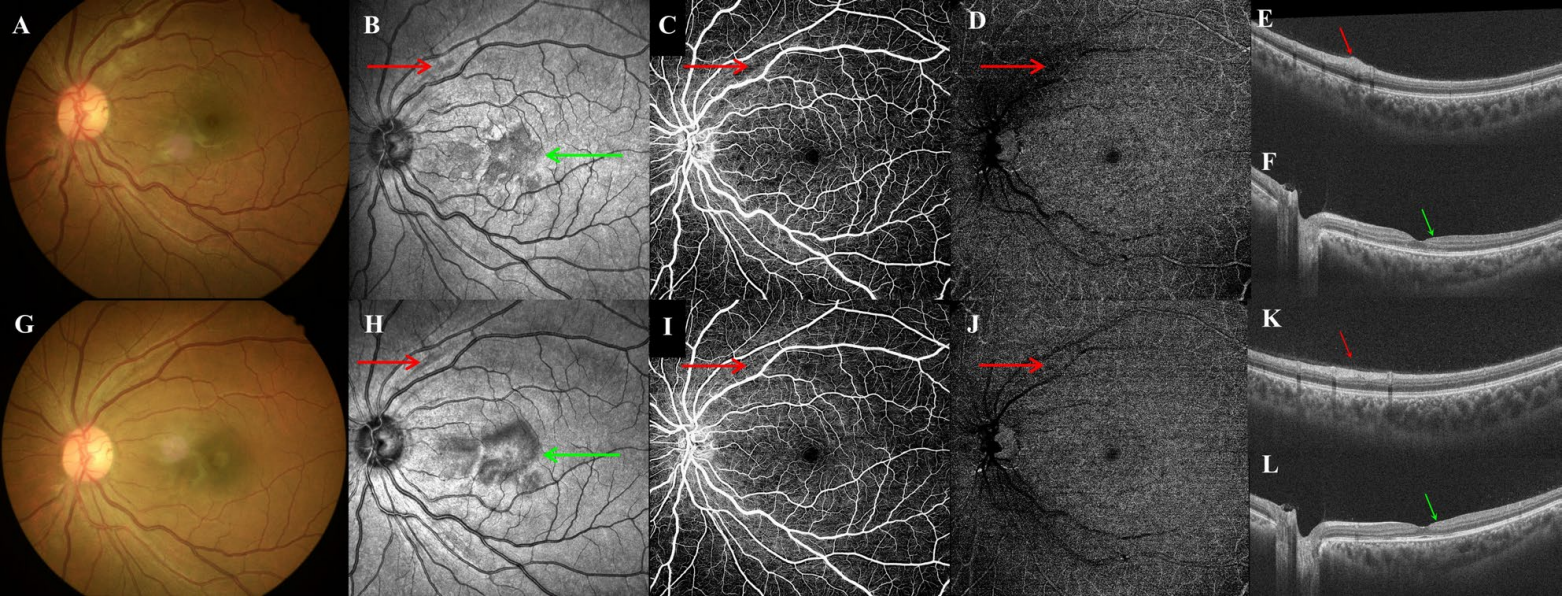
Multimodal imaging study of acute macular neuroretinopathy. Patient No. 86 (**A**–**L**): combined AMN and Purtscher-like retinopathy. Fundus photography (**A**) shows multiple cotton-wool spots, whereas near-infrared imaging (**B**, red arrow) and SS-OCT (**E**, red arrow) demonstrate edema in the retinal nerve fiber layer. OCTA-SCP (**C**, red arrow) shows a shielding signal caused by cotton-wool spots. OCTA-DCP (**D**, red arrow) shows a more distinct shielding signal than SCP. NIR (**B**, green arrow) of the left eye shows irregular hyporeflective areas at the macula and SS-OCT (**F**, green arrow) shows hyperreflectivity at the level of the outer plexiform and outer nuclear layers, corresponding to the AMN lesion. At the 1-month follow-up of the lesion sites, fundus photography (**G**) and OCT angiography-SCP (**I**, red arrow) and DCP (**J**, red arrow) show fading or resolution of multiple cotton-wool spots. NIR (**H**, red arrow) and SS-OCT (**K**, red arrow) demonstrate reduced edema in the retinal nerve fiber layer. In SS-OCT (**L**, green arrow), hyperreflective lesions in the OPL and ONL are resolved, with EZ areas exhibiting partial recovery. AMN, acute macular neuroretinopathy; SS-OCT, swept-source optical coherence tomography; OCTA, optical coherence tomography angiography; SCP, superficial capillary plexus; DCP, deep capillary plexus; NIR, near-infrared imaging; OPL, outer plexiform layer; ONL, outer nuclear layer; EZ, ellipsoid zone

The rapid increase in COVID-19-related AMN has sparked new discussions regarding its underlying mechanisms. Despite some controversies, associations have been reported between AMN and retinal or choroid intravascular flow velocity, blood volume variation, and vascular impairment [13, 14]. Some factors may be associated with COVID-19-related AMN. COVID-19 increases the risk of cytokine storms and thrombotic complications [15], exacerbating inflammation, coagulation dysfunction, and vascular endothelial damage [16]. A postmortem analysis detected severe acute respiratory syndrome coronavirus-2 RNA in retinal tissues, accompanied by abnormalities suggestive of indirect viral effects, including cytoid bodies, microvascular changes in the retina and choroid, retinal edema, and minimal inflammation [17]. These findings suggest that inflammation and hypercoagulation in retinal and choroidal vessels may contribute to the pathological mechanisms of AMN. Notably, during the COVID-19 pandemic, we also observed many patients with Purtscher-like retinopathy. In our case data list, two cases (patient Nos. 48, 86) had Purtscher-like retinopathy combined with AMN (Fig. 2A–L, multimodal imaging of patient No. 86). Previously published studies have attributed Purtscher-like retinopathy to the microembolization of retinal arterioles and choroidal hypoperfusion [18, 19]. Based on monistic considerations, AMN in this case may also have been caused by circulatory compromise in variant retinal and choroid vascular layers.

In our cohort, 76% (*n* = 68) of patients experienced high fever (≥ 38.6 °C), 14% (*n* = 13) had mild fever (37.5–38.5 °C), and 46% (*n* = 41) reported profuse diaphoresis secondary to high fever. Such fever-related dehydration, inferred from clinical symptoms rather than objective laboratory measurements, may lead to vascular hypoperfusion and oxygen deficiency, which could also be a possible cause of AMN. A previous study suggested that AMN lesions occur in the most ischemia-sensitive region of the retina [20].

Currently, there is no evidence-based or universally accepted treatment guideline for acute macular neuroretinopathy (AMN). Most reported cases are managed conservatively with observation and supportive care [5, 6], while therapeutic interventions, such as systemic corticosteroids, have been reported only sporadically in isolated cases or small case series, with variable and inconsistent outcomes [21]. In the absence of controlled studies and standardized treatment protocols, conservative follow-up remains the most adopted management approach in clinical practice. In our cohort, among the five clinic-followed patients, four received periocular triamcinolone acetonide injections based on clinical judgment, whereas one patient presented approximately one month after symptom onset and was therefore managed conservatively treatment only, given the limited expected benefit of periocular steroid therapy at that stage. For the online cohort, treatment-related information was obtained only on a voluntary basis through patient self-report and uploaded medical records. Among the cases in which treatment information was available, most patients reported receiving steroid-based therapy at local institutions.

Despite our efforts to gather comprehensive information on the patients, this study has inherent limitations. First, this was a descriptive study without a randomized control group. Second, patient data were predominantly collected through an internet survey supplemented by personal interviews. Basic information, as well as subjective feelings related to AMN symptoms, were self-reported by patients through online question and answer sessions. Ophthalmic testing results (visual acuity and OCT) and COVID-19 vaccination status were obtained from the local hospitals where the patients received treatment. To reduce bias, we excluded patients with low-quality ophthalmic imaging and provided the Amsler grid online, both at presentation and during follow-up, to assess visual disturbances. As treatment was not considered a core exposure variable in the present study, AMN-related therapeutic strategies and treatment–prognosis comparisons were not systematically collected or analyzed.

All followed-up patients underwent OCT examinations. However, because OCT data for many patients were obtained via online submission of screenshots or B-scan images rather than original volumetric data, lesion presence and foveal involvement could be reliably assessed but data formats precluded detailed quantitative analyses. In the follow-up analysis, quantitative risk analysis for poor BCVA recovery was not performed, and potential prognostic factors were discussed qualitatively only.

In conclusion, our study presents the largest case series to date on COVID-19-related AMN, comprising a comprehensive analysis of 152 eyes from 90 patients, with 96 eyes from 56 patients who had follow-up data for an average duration of 9 months. This substantial dataset offers a detailed understanding of the epidemiological, clinical, and prognostic features associated with this condition. However, the underlying mechanism of COVID-19-related AMN warrants further exploration. Factors such as hyperinflammatory and hypercoagulable states in retinal and choroidal circulation, as well as vascular hypoperfusion following fever-induced dehydration, may contribute to the development of this condition. Overall, our study highlights the importance of ophthalmologist vigilance for COVID-19-related retinal complications, particularly AMN. Follow-up is crucial for monitoring the progression and evolution of visual symptoms and retinal changes over time.

## Supplementary Information

Below is the link to the electronic supplementary material.

Supplementary Material 1

## Data Availability

The data that support the findings of this study are available from the corresponding author, JD, upon reasonable request.

## Abbreviations

AMN: Acute macular neuroretinopathy
COVID-19: Coronavirus disease 2019
OCT: Optical coherence tomography
EZ: Ellipsoid zone
SD-OCT: Spectral-domain OCT
BCVA: Best-corrected visual acuity
