# Supplementary material for: COVID-19-related acute macular neuroretinopathy in China: a retrospective study of 90 cases (152 eyes)
Source: Int J Retina Vitreous. 2026 Feb 28;12:55. doi: 10.1186/s40942-026-00825-2 (PMC13059550; doi:10.1186/s40942-026-00825-2)
Supplement: Supplementary file 1 — Supplementary Material 1 [file 40942_2026_825_MOESM1_ESM.pdf]

| Number | Gender (Female) or (Male) | Age | Side | Baseline BCVA (COVID-19 INFECTED) | Body temperature | Take antipyretics (Ture) or not(False) | Accompanying symptoms | Profuse diaphoresis secondary to high fever | Influenza-like symptoms to visual symptoms interval (Day) | Ocular symptoms of AMN | Drug history | Concurrent risk factors | COVID-19 vaccinated | Adverse reactions after vaccine injection | Fovea invloved on initial OCT (OD) | Fovea invloved on initial OCT (OS) | Follow-up duration (months) | Final BCVA (FOLLOW-UP) | Additional symptoms during follow-up | Prognosis of ocular symptoms of AMN |
|---|---|---|---|---|---|---|---|---|---|---|---|---|---|---|---|---|---|---|---|---|
| 1 | F | 34 | OU | 20/30 OD,20/40 OS | 40.0 | T | Dizzy | T | 2 | Black dots OD,Central scintillating scotomas OS | No | LASIK,Gestational hypertension,Caesarean twice | T | T(episodic hypertension） | T | T | 5.9 | 20/25 OD,20/25 OS | Frequent ocular fatigue, visual snow | Improved |
| 2 | F | 30 | OU | 20/25 OU | 37.6 | T | Headache | F | 1 | Central mosaic blurred vision OU | Transamin 0.5mg qd | yellow-brown spots on face | F |  | T | T | 3.0 | 20/20 OD,20/20 OS | Visual snow | Unchanged |
| 3 | F | 32 | OU | 20/25 OD,20/100 OS | 39.1 | F | No | F | 2 | Paracentral scotoma OU | No | Caesarean once | T | F | T | T | 11.0 | 20/25 OD,20/100 OS | Water ripples | Unchanged |
| 4 | F | 24 | OU | 20/25 OU | 39.6 | T | Eye Pain | F | 2 | Paracentral scotoma OD,Paracentral scotoma with water wave pattern OS | No | Galactoma | T | F | F | F | 3.0 | 20/20 OD,20/20 OS | Frequent ocular fatigue, water ripples | Improved |
| 5 | F | 36 | OU | 20/30 OU | 37.8 | F | No | F | 2 | Paracentral scotoma OU | Enzyme | Traumatic fracture | T | F | T | T | 3.0 | 20/30 OD,20/30 OS | Floaters | Unchanged |
| 6 | F | 26 | OU | 20/200 OU | Unchecked | T | Eye Swelling | F | 2 | Paracentral scotoma OU | No | Breast fibromas | T | F | T | T | 3.0 | 20/100 OD,20/100 OS | No secondary ocular symptoms | Improved |
| 7 | F | 24 | OS | 20/20 OS | 38.0 | T | Headache | F | 3 | Paracentral mosaic blurred vision OS | No | No | T | F |  | T | 0.0 |  |  |  |
| 8 | F | 35 | OU | 20/20 OD,20/40 OS | 37.5 | T | Headache | T | 3 | Paracentral scotoma OD,Central scotoma OS | No | No | F |  | T | T | 15.0 | 20/20 OD,20/20 OS | Floaters | Improved |
| 9 | F | 20 | OU | 20/25 OD,20/100 OS | Unchecked | T | Headache | F | 2 | Mosaic blurred vision OU | Amoxicillin | Traumatic fracture | T | F | F | T | 0.0 |  |  |  |
| 10 | F | 24 | OS | 20/20 OS | 39.0 | T | Headache | T | 1 | Paracentral scintillating scotomas OS | No | No | F |  |  | F | 0.0 |  |  |  |
| 11 | F | 27 | OU | 20/20 OU | 39.0 | T | Headache | F | 2 | Mosaic blurred vision OU | Sodium Hyaluronate Eye Drops | Dry eye syndrome | T | F | F | T | 0.0 |  |  |  |
| 12 | F | 35 | OU | 20/20 OU | 39.1 | T | Headache,Dizzy | F | 2 | Central mosaic blurred vision OU | Antihypertensive drugs | High blood pressure,Periocular operation | T | F | T | T | 13.6 | 20/20 OD,20/20 OS | Frequent ocular fatigue | Unchanged |
| 13 | F | 25 | OU | 20/25 OD,20/20 OS | 39.1 | T | No | F | 2 | Mosaic blurred vision OU | No | Double eyelidplasty, High myopia | T | F | T | F | 3.0 | 20/20 OD,20/20 OS | Water ripples | Improved |
| 14 | F | 32 | OU | 20/20 OU | Unchecked | F | Headache | F | 2 | Mosaic blurred vision OU | No | No | T | F | T | T | 1.0 |  |  |  |
| 15 | F | 31 | OU | 20/20 OU | 38.5 | T | headache, Eriorbital pain | F | 1 | Central scintillating scotomas OU | No | No | T | F | T | T | 11.5 | 20/20 OD,20/20 OS | Frequent ocular fatigue, water ripples | Improved |
| 16 | M | 20 | OU | Unknow | 39.0 | T | Headache | T | 2 | Mosaic blurred vision OU | Hydroxychloroquine Sulfate Tablets,Valsartan | Chronic nephritis | T | F | T | T | 0.0 |  |  |  |
| 17 | F | 26 | OU | Unknow | 39.1 | / | Headache | F | 2 | Sporadic scotomas OS | No | No | F |  | T | T | 0.0 |  |  |  |
| 18 | F | 24 | OU | 20/20 OD,20/40 OS | 39.8 | T | No | F | 2 | Central scintillating scotomas OU | No | No | T | F | F | F | 2.0 |  |  |  |
| 19 | F | 25 | OS | 20/20 OS | 39.2 | T | Headache,Eye Pain | F | 3 | Central scintillating scotoma OS | No | No | F |  |  | F | 0.0 |  |  |  |
| 20 | F | 30 | OU | 20/20 OU | 40.0 | T | No | T | 2 | Central scintillating scotomas OU | No | Adenoidectomy | T | F | T | T | 14.0 | 20/20 OD,20/20 OS | Frequent ocular fatigue, floaters,water ripples | Worse |
| 21 | F | 25 | OS | 20/20 OS | 40.0 | T | Headache | T | 3 | Central scintillating scotoma OS | No | No | T | F |  | T | 1.0 |  |  |  |
| 22 | F | 25 | OU | 20/20 OU | 39.0 | T | Headache | T | 2 | Central scintillating scotomas OU | Contraceptive drugs, Anti-inflammatory drugs | No | T | F | T | T | 15.0 | 20/20 OD,20/20 OS | Frequent ocular fatigue | Unchanged |
| 23 | F | 25 | OD | 20/40 OD | 39.5 | T | Eye Pain | T | 3 | Central scotomas OD | No | Floaters | T | F | T |  | 1.0 |  |  |  |
| 24 | F | 29 | OS | 20/25 OS | 39.4 | T | Headache | T | 3 | Central scotomas OS | Salbutamol (inhaled) | Asthma,LASIK | T | F |  | T | 15.0 | 20/20 OS | Frequent ocular fatigue, Floaters,water ripples | Improved |

| Number | Gender (Female) or (Male) | Age | Side | Baseline BCVA (COVID-19 INFECTED) | Body temperature | Take antipyretics (Ture) or not(False) | Accompanying symptoms | Profuse diaphoresis secondary to high fever | Influenza-like symptoms to visual symptoms interval (Day) | Ocular symptoms of AMN | Drug history | Concurrent risk factors | COVID-19 vaccinated | Adverse reactions after vaccine injection | Fovea invloved on initial OCT (OD) | Fovea invloved on initial OCT (OS) | Follow-up duration (months) | Final BCVA (FOLLOW-UP) | Additional symptoms during follow-up | Prognosis of ocular symptoms of AMN |
|---|---|---|---|---|---|---|---|---|---|---|---|---|---|---|---|---|---|---|---|---|
| 25 | M | 26 | OS | 20/20 OS | 38.8 | T | Headache | F | 3 | Paracentral scintillating scotoma OS | No | No | T | F | | F | 3.0 | 20/20 OS | Frequent ocular fatigue, floaters | Unchanged |
| 26 | F | 22 | OU | 20/400 OU | 38.5 | T | No | F | 4 | Central scotomas OU | No | Gestational high blood pressure,Recent cesarean operation | T | F | T | T | 4.0 | 20/30 OD,20/30 OS | Water ripples | Improved |
| 27 | M | 38 | OD | 20/20 OD | 39.3 | T | Headache,Right Eye Swelling | T | 2 | Paracentral scotoma OD | No | No | T | F | T | | 1.0 | | | |
| 28 | F | 22 | OS | 20/20 OS | 40.8 | T | No | T | 3 | Central scintillating scotoma with water wave pattern OS | No | Ocular hypertension | T | F | | T | 3.0 | 20/20 OS | Water ripples | Unchanged |
| 29 | F | 23 | OU | 20/20 OU | 38.3 | T | Headache,Eye Pain | T | 3 | Paracentral mosaic blurred vision OU | No | No | T | F | T | F | 1.0 | | | |
| 30 | F | 33 | OU | 20/20 OU | 38.7 | T | No | F | 3 | Paracentral scotomas OU | No | No | F | | T | F | 0.5 | | | |
| 31 | F | 33 | OU | 20/30 OD,20/50 OS | 38.5 | T | Headache,Eye Pain,Photophobia | T | 2 | Scotomas OU | Toremifene Citrate Tablets,Leuprorelin injection | Wide local excision of breast carcinoma | T | F | T | T | 1.0 | | | |
| 32 | F | 23 | OU | 20/200 OD,20/25 OS | 39.0 | T | Headache | T | 2 | Mosaic blurred vision OU | No | Periocular injection | T | F | T | T | 5.0 | 20/50 OD,20/25 OS | Frequent ocular fatigue | Unchanged |
| 33 | M | 20 | OU | 20/20 OD,20/40 OS | 39.0 | T | No | F | 3 | Paracentral scintillating scotomas OU | Antiviral drug | No | T | F | T | T | 11.0 | 20/20 OD,20/30 OS | Water ripples | Improved |
| 34 | F | 23 | OU | 20/400 OU | 39.0 | T | No | T | 2 | Central scintillating scotomas OU | No | Hypotension,Migraine | T | F | T | T | 1.0 | | | |
| 35 | M | 28 | OU | 20/20 OU | 38.6 | T | Headache | F | 3 | Paracentral mosaic blurred vision OU | No | Migraine | T | F | F | T | 14.0 | 20/20 OD,20/20 OS | Water ripples | Worse |
| 36 | M | 23 | OU | 20/20 OU | 40.0 | T | Headache | T | 2 | Sporadic scintillating scotoma OU | No | Genetic high blood pressure | T | F | T | T | 1.0 | | | |
| 37 | F | 24 | OU | 20/20 OU | 39.0 | T | Headache,Tears | T | 3 | Paracentral scintillating scotomas OU | No | No | T | F | T | T | 1.0 | | | |
| 38 | M | 34 | OU | 20/40 OD,20/30 OS | 39.5 | T | Headache | T | 4 | Central mosaic blurred vision OU | No | High blood pressure | T | F | T | F | 3.3 | 20/30 OD,20/25 OS | Water ripples | Improved |
| 39 | F | 30 | OD | 20/25 OD | 40.0 | T | No | F | 2 | Paracentral mosaic blurred vision OD | No | No | F | | T | | 15.0 | 20/25 OD | Water ripples | Unchanged |
| 40 | F | 36 | OU | 20/100 OD,20/200 OS | 39.2 | T | No | F | 3 | Sporadic mosaic blurred vision OU | No | Iritis,Acute cholecystitis | T | F | T | T | 9.0 | 20/100 OD,20/100 OS | Floaters | Unchanged |
| 41 | F | 23 | OU | 20/200 OD,20/200 OS | 39.7 | / | No | T | 3 | Central mosaic blurred vision OU | Contraceptive drugs(Yasmin) for one month | Migraine, Hashimoto thyroiditis | F | | T | T | 15.0 | 20/25 OD,20/25 OS | Water ripples | Improved |
| 42 | F | 18 | OU | 20/30 OD,20/25 OS | 39.0 | T | Headache,Eye Pain | T | 3 | Sporadic mosaic blurred vision OU | / | / | F | | T | T | 1.0 | | | |
| 43 | M | 28 | OS | 20/20 OS | 40.0 | T | Headache,Eye Pain | T | 2 | Paracentral scotomas OS | / | / | T | F | | F | 0.0 | | | |
| 44 | M | 21 | OS | 20/20 OS | 39.0 | T | Headache | T | 3 | Paracentral mosaic blurred vision OS | No | No | T | F | | T | 0.0 | | | |
| 45 | F | 32 | OU | 20/20 OD,20/40 OS | 37.8 | T | Headache | F | 2 | Paracentral scotomas OU | No | Induced abortion | T | F | T | T | 15.0 | 20/20 OD,20/25 OS | No secondary ocular symptoms | Worse |
| 46 | F | 17 | OS | 20/30 OS | 39.8 | T | Headache | T | 2 | Central mosaic blurred vision OS | No | No | T | F | | T | 0.0 | | | |
| 47 | F | 23 | OU | 20/40 OD,20/30 OS | 38.6 | T | Headache | T | 2 | Central scotomas OU | No | Conjunctivitis,Trichiasis | T | F | T | T | 7.5 | 20/20 OD,20/25 OS | Floaters | Unchanged |
| 48 | M | 32 | OS | 20/20 OS | 40.0 | T | Headache,Eye Pain | T | 2 | Central scotomas OS | / | / | F | | | T | 6.5 | 20/20 OS | No secondary ocular symptoms | Unchanged |
| 49 | F | 34 | OD | 20/100 OD | 39.6 | T | Headache | F | 3 | Central mosaic blurred vision OD | Hypotensive drugs | High blood pressure | T | T (Scattered rash on the face and trunk) | T | | 3.0 | 20/50 OD | / | Unchanged |
| 50 | F | 32 | OU | 20/200 OU | 39.0 | T | Headache | T | 2 | Sporadic mosaic blurred vision OU | Bromocriptine | Kidney failure | F | | T | T | 7.0 | 20/100 OD,20/100 OS | Floaters | Unchanged |

| Number | Gender (Female) or (Male) | Age | Side | Baseline BCVA (COVID-19 INFECTED) | Body temperature | Take antipyretics (Ture) or not(False) | Accompanying symptoms | Profuse diaphoresis secondary to high fever | Influenza-like symptoms to visual symptoms interval (Day) | Ocular symptoms of AMN | Drug history | Concurrent risk factors | COVID-19 vaccinated | Adverse reactions after vaccine injection | Fovea invloved on initial OCT (OD) | Fovea invloved on initial OCT (OS) | Follow-up duration (months) | Final BCVA (FOLLOW-UP) | Additional symptoms during follow-up | Prognosis of ocular symptoms of AMN |
|---|---|---|---|---|---|---|---|---|---|---|---|---|---|---|---|---|---|---|---|---|
| 51 | F | 38 | OU | 20/25 OD,20/200 OS | 39.5 | T | Headache | F | 2 | Central scotomas OU | No | No | T | F | T | T | 3.6 | 20/30 OD,20/100 OS | Water ripples | Worse |
| 52 | F | 28 | OU | 20/200 OU | 39.6 | T | Headache,Eye Pain | T | 2 | Paracentral mosaic blurred vision OU | Iron supplementation | Chronic anemia | T | F | F | T | 1.0 | | | |
| 53 | F | 36 | OU | 20/20 OD,20/100 OS | 39.4 | T | No | F | 3 | Paracentral scotomas OU | No | No | T | F | T | T | 12.0 | 20/20 OD,20/100 OS | No secondary ocular symptoms | Unchanged |
| 54 | F | 32 | OS | 20/200 OS | 39.4 | T | Headache | F | 3 | Paracentral scotomas OS | No | No | T | F | | T | 15.0 | 20/200 OS | Frequent ocular fatigue,floaters, water ripples | Unchanged |
| 55 | F | 32 | OU | 20/100 OD,20/200 OS | 39.5 | T | Headache,Eye Pain, Photophobia,Tears | F | 3 | Central scotomas OU | No | No | T | F | T | T | 0.5 | | | |
| 56 | F | 37 | OU | 20/50 OU | 38.2 | T | Headache | F | 3 | Paracentral scotomas OU | No | Caesarean once | T | F | T | T | 1.0 | | | |
| 57 | M | 48 | OS | 20/50 OS | 38.4 | T | Headache | F | 2 | Mosaic blurred vision OS | No | Blurred vision in right eye | T | F | | T | 3.0 | 20/25 OS | / | Improved |
| 58 | F | 29 | OS | 20/30 OS | 39.6 | T | Headache | T | 2 | Mosaic blurred vision OS | Emergency contraception one month ago | High blood pressure,Migraine, Caesarean,Cholecystectomy | T | F | | T | 12.0 | 20/30 OS | Water ripples | Unchanged |
| 59 | F | 25 | OU | 20/20 OU | 38.6 | T | Headache | F | 2 | Paracentral scotomas OU | No | No | T | F | T | T | 5.6 | 20/20 OD,20/20 OS | No secondary ocular symptoms | Unchanged |
| 60 | M | 35 | OS | 20/20 OS | 39.6 | T | Headache, Eye Swelling,Eye Movement Pain | F | 2 | Central mosaic blurred vision OS | No | Asthenopia | T | F | | T | 0.0 | | | |
| 61 | F | 32 | OD | 20/20 OD | 39.6 | T | Headache, Eye Movement Pain | F | 3 | Central scotomas OD | No | Allergic constitution | T | F | T | | 10.6 | 20/20 OD | Floaters, water ripples | Improved |
| 62 | F | 37 | OU | 20/25 OD,20/30 OS | Unchecked | T | No | F | 2 | Mosaic blurred vision OU | / | Chronic nephritis (IgA nephropathy) | F | | T | T | 1.0 | | | |
| 63 | F | 27 | OU | 20/20 OU | Unchecked | / | No | F | 2 | Scotomas OU | / | / | F | | F | T | 2.0 | | | |
| 64 | F | 36 | OS | 20/200 OS | 38.5 | T | Headache | F | 2 | Mosaic blurred vision OD,Central scotomas OS | No | No | T | F | | T | 1.0 | | | |
| 65 | M | 14 | OS | 20/60 OS | 38.6 | T | No | F | 2 | Scotomas OS | No | No | T | F | | T | 0.0 | | | |
| 66 | F | 24 | OS | 20/20 OS | 40.3 | / | / | T | 1 | Scotoma OS | No | Ocular hypertension | F | | | T | 5.0 | 20/20 OS | No secondary ocular symptoms | Unchanged |
| 67 | F | 22 | OU | 20/60 OD,20/30 OS | 39.7 | F | No | T | 2 | Paracentral scintillating scotoma OD,Paracentral scotomas with water wave pattern OS | No | Tonsillectomy | T | F | T | T | 13.0 | 20/50 OD,20/25 OS | Water ripples | Unchanged |
| 68 | F | 34 | OU | 20/30 OD,20/25 OS | 39.0 | T | / | T | 2 | Paracentral scotomas OU | No | No | T | F | T | T | 9.0 | 20/40 OD,20/40 OS | Water ripples | Worse |
| 69 | F | 33 | OU | 20/20 OU | 39.0 | F | No | F | 2 | Paracentral scotomas OU | No | Dry eye syndrome, Double eyelidplasty | T | T(headche,fatigue) | T | F | 1.0 | | | |
| 70 | F | 25 | OU | 20/200 OD,20/60 OS | 38.6 | T | Headache | T | 2 | Scotomas OU | No | No | T | T(painful edematous plaques in the injection site） | F | F | 15.0 | 20/20 OD,20/20 OS | Floaters | Improved |
| 71 | F | 33 | OU | 20/20 OU | 39.0 | T | Foreign body sensation in the eye | T | 2 | Paracentral scintillating scotomas OU | Contraceptive drugs for 3 weeks | Anxiety Disorder | T | F | F | F | 14.0 | 20/20 OD,20/20 OS | Floaters | Improved |
| 72 | F | 29 | OU | 20/20 OD,20/25 OS | 40.0 | T | headache,Orbital pain | T | 3 | Mosaic blurred vision OU | TobraDex eye drops | Myocardial ischemia, Bradycardia | T | T（Scattered rash on the face and trunk, [illegible] | T | T | 3.0 | 20/20 OD,20/20 OS | Frequent ocular fatigue, water ripples | Unchanged |
| 73 | M | 36 | OU | 20/20 OU | 40.0 | T | No | T | 3 | Paracentral mosaic blurred vision OU | No | No | T | T(mild fever for 2 weeks) | T | T | 14.0 | 20/20 OD,20/30 OS | Frequent ocular fatigue, water ripples | Worse |
| 74 | F | 34 | OU | 20/20 OU | 39.0 | F | Tears | F | 2 | mosaic blurred vision OU | Thyrozol for one year | Inherited ocular hypertension, Hyperthyroidism | T | F | F | F | 4.0 | 20/20 OD,20/20 OS | Water ripples | Improved |
| 75 | M | 29 | OU | 20/20 OD,20/25 OS | 39.0 | T | Severe Headache | F | 3 | Paracentral scotomas OD,Central and paracentral scotomas OS | No | No | F | | T | T | 1.0 | | | |
| 76 | M | 31 | OU | 20/60 OD,20/200 OS | 41.0 | T | Headache | F | 2 | Central scotomas OU | No | No | T | F | T | T | 12.0 | 20/30 OD,20/30 OS | Water ripples | Improved |

| Number | Gender (Female) or (Male) | Age | Side | Baseline BCVA (COVID-19 INFECTED) | Body temperature | Take antipyretics (Ture) or not(False) | Accompanying symptoms | Profuse diaphoresis secondary to high fever | Influenza-like symptoms to visual symptoms interval (Day) | Ocular symptoms of AMN | Drug history | Concurrent risk factors | COVID-19 vaccinated | Adverse reactions after vaccine injection | Fovea invloved on initial OCT (OD) | Fovea invloved on initial OCT (OS) | Follow-up duration (months) | Final BCVA (FOLLOW-UP) | Additional symptoms during follow-up | Prognosis of ocular symptoms of AMN |
|---|---|---|---|---|---|---|---|---|---|---|---|---|---|---|---|---|---|---|---|---|
| 77 | M | 24 | OS | 20/40 OS | 38.6 | T | No | F | 3 | Paracentral scintillating scotoma OS | No | No | T | F | | F | 3.0 | 20/30 OS | Water ripples | Improved |
| 78 | M | 20 | OU | 20/20 OU | 38.6 | T | Headache | F | 1 | Scotomas OD,Black dots OS | No | No | F | | F | F | 12.3 | 20/20 OD,20/20 OS | No secondary ocular symptoms | Unchanged |
| 79 | F | 24 | OU | 20/25 OD,20/40 OS | 40.0 | T | Headache, Severe eye Pain | F | 3 | Paracentral scintillating scotomas OU | No | Anemia | F | | F | F | 3.6 | 20/25 OD,20/25 OS | Frequent ocular fatigue,water ripples | Unchanged |
| 80 | F | 26 | OU | 20/25 OD,20/40 OS | Unchecked | / | / | F | 2 | Scotomas OU | No | No | F | | T | T | 3.0 | 20/20 OD,20/20 OS | No secondary ocular symptoms | Improved |
| 81 | F | 32 | OU | 20/20 OU | Unchecked | / | / | F | 2 | Black dots OU | No | No | F | | F | T | 12.2 | 20/20 OD,20/20 OS | Floaters | Unchanged |
| 82 | M | 46 | OU | 20/40 OD,20/25 OS | Unchecked | / | / | F | 2 | Mosaic blurred vision OU | No | No | F | | T | T | 1.0 | | | |
| 83 | F | 27 | OU | 20/40 OU | 39.0 | / | / | T | 2 | Scintillating scotomas OU | Amlodipine tablet(Hypotensive drugs),Roxastat capsules（Improve anemia）for 2 years | Chronic renal failure,Renal high blood pressure,Renal anemia | F | | T | T | 1.5 | | | |
| 84 | F | 28 | OD | 20/25 OD | Unchecked | / | Eye Pain | F | 2 | Scotomas OD | No | No | F | | T | | 15.0 | 20/20 OD | Floaters | Worse |
| 85 | M | 27 | OU | 20/40 OD,20/25 OS | 40.0 | T | Photophobia | T | 3 | Scotomas OU | No | Floaters | F | | F | F | 12.0 | 20/20 OD,20/25 OS | No secondary ocular symptoms | Improved |
| 86 | F | 26 | OU | 20/50 OD,20/30 OS | 39.0 | T | Severe eye pain | T | 1 | Scotomas OU | No | No | T | F | T | T | 12.0 | 20/25 OD,20/20 OS | Visual snow | Improved |
| 87 | F | 31 | OD | 20/40 OD | 39.0 | T | No | T | 1 | Mosaic blurred vision OD | No | No | T | F | T | | 12.0 | 20/25 OD | Frequent ocular fatigue | Unchanged |
| 88 | F | 32 | OS | 20/30 OS | 39.8 | T | Headache,Eye Pain,Tears,Eye Secretion Increased | T | 2 | Scotomas OS | Emergency contraception 3 month ago | No | T | F | | T | 15.0 | 20/40 OS | Water ripples | Unchanged |
| 89 | F | 26 | OD | 20/25 OD | 39.5 | T | Dizzy | T | 1 | Scintillating scotoma OD | No | No | T | F | T | | 6.2 | 20/20 OD | Floaters | Improved |
| 90 | F | 25 | OU | 20/30 OD,20/100 OS | 38.5 | T | Eye Pain | F | 1 | Mosaic blurred vision OU | No | No | T | F | T | T | 15.0 | 20/30 OD,20/30 OS | Water ripples | Unchanged |

**Table footnote**
Side: OD, oculus dexter; OS, oculus sinister; OU, oculus uterque.
The following columns:Take antipyretics, Profuse diaphoresis secondary to high fever, COVID-19 vaccinated, Adverse reactions after vaccine injection,Fovea invloved on initial OCT (OD),Fovea invloved on initial OCT (OD): T-True, F-False.
"/" indicates that the corresponding patient information was not collected.
Blank entries in the follow-up section indicate non-participation in follow-up.
